# 5,5′-Diethyl-2,2′-(triazene-1,3-di­yl)di-1,3,4-thia­diazole

**DOI:** 10.1107/S1600536808033941

**Published:** 2008-10-22

**Authors:** Hai-Su Zeng, Lu-Na Han, Si-shun Kang, Hai-lin Li, Hai-bo Wang

**Affiliations:** a College of Science, Nanjing University of Technology, Xinmofan Road No. 5 Nanjing, Nanjing 210009, People’s Republic of China

## Abstract

In the mol­ecule of the title compound, C_8_H_11_N_7_S_2_, the conformation about the N=N bond is *trans* and the thia­diazole rings are oriented at a dihedral angle of 2.92 (3)°. In the crystal structure, inter­molecular N—H⋯S hydrogen bonds link the mol­ecules into chains. There are π–π contacts between the thia­diazole rings [centroid-to-centroid distances = 3.699 (3) and 3.720 (2) Å].

## Related literature

For general background, see: Bach *et al.* (1996 ▶); Clark & Hester (1991 ▶); Taniike *et al.* (1996 ▶). For bond-length data, see: Allen *et al.* (1987 ▶).
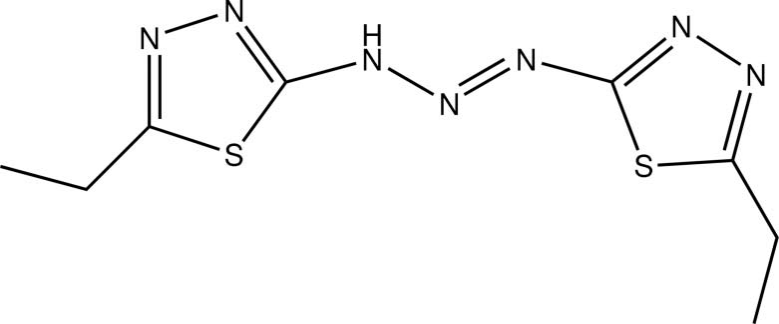


## Experimental

### 

#### Crystal data

C_8_H_11_N_7_S_2_
*M* *_r_* = 269.36
Monoclinic, 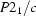

*a* = 12.188 (2) Å
*b* = 9.1460 (18) Å
*c* = 12.790 (3) Å
β = 110.99 (3)°
*V* = 1331.1 (5) Å^3^
*Z* = 4
Mo *K*α radiation
μ = 0.39 mm^−1^
*T* = 294 (2) K
0.20 × 0.10 × 0.10 mm

#### Data collection

Enraf–Nonius CAD-4 diffractometer
Absorption correction: ψ scan (North *et al.*, 1968 ▶) *T*_min_ = 0.926, *T*_max_ = 0.962
2431 measured reflections
2320 independent reflections
1468 reflections with *I* > 2σ(*I*)
*R*_int_ = 0.0047
3 standard reflections frequency: 120 min intensity decay: none

#### Refinement

*R*[*F* ^2^ > 2σ(*F* ^2^)] = 0.072
*wR*(*F* ^2^) = 0.178
*S* = 1.00
2320 reflections
142 parameters
H-atom parameters constrained
Δρ_max_ = 0.62 e Å^−3^
Δρ_min_ = −1.06 e Å^−3^

### 

Data collection: *CAD-4 Software* (Enraf–Nonius, 1989 ▶); cell refinement: *CAD-4 Software*; data reduction: *XCAD4* (Harms & Wocadlo, 1995 ▶); program(s) used to solve structure: *SHELXS97* (Sheldrick, 2008 ▶); program(s) used to refine structure: *SHELXL97* (Sheldrick, 2008 ▶); molecular graphics: *ORTEP-3 for Windows* (Farrugia, 1997 ▶) and *PLATON* (Spek, 2003 ▶); software used to prepare material for publication: *SHELXTL* (Sheldrick, 2008 ▶).

## Supplementary Material

Crystal structure: contains datablocks global, I. DOI: 10.1107/S1600536808033941/hk2554sup1.cif

Structure factors: contains datablocks I. DOI: 10.1107/S1600536808033941/hk2554Isup2.hkl

Additional supplementary materials:  crystallographic information; 3D view; checkCIF report

## Figures and Tables

**Table 1:** Hydrogen-bond geometry (Å, °)

*D*—H⋯*A*	*D*—H	H⋯*A*	*D*⋯*A*	*D*—H⋯*A*
N5—H5*A*⋯S2^i^	0.86	2.84	3.631 (4)	154

Symmetry code: (i) 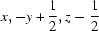.

**Fig. 1.**
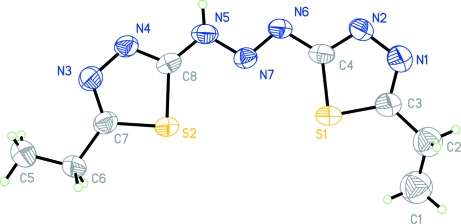
The molecular structure of the title molecule, with the atom-numbering scheme. Displacement ellipsoids are drawn at the 30% probability level.

**Fig. 2.**
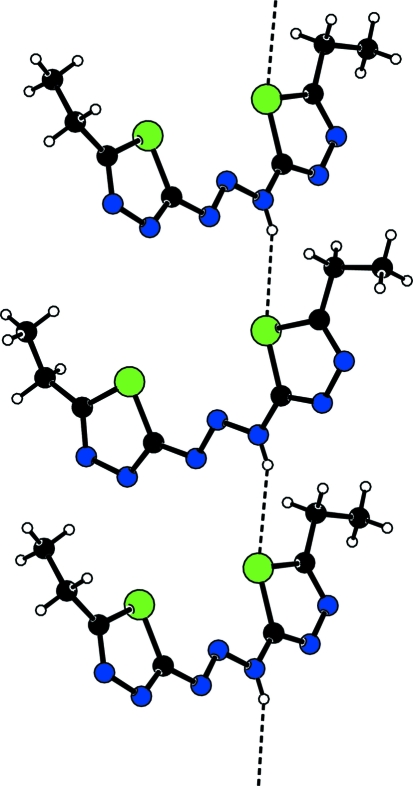
A partial packing diagram of the title compound.

## supplementary crystallographic information

### Comment

The photophysical properties of azo compounds are of large interest in the development of nonlinear optical and optical data storage materials (Bach *et al.*, 1996; Taniike *et al.*, 1996; Clark & Hester, 1991). As part of our studies in this area, we report herein the synthesis and crystal structure of the title compound.

In the title compound (Fig. 1), the bond lengths (Allen *et al.*, 1987) and angles are within normal ranges. Rings A (S1/N1/N2/C3/C4) and B (S2/N3/N4/C7/C8) are oriented at a dihedral angle of 2.92 (3)°. So, they are nearly coplanar.

In the crystal structure, intermolecular N—H···S hydrogen bonds (Table 1) link the molecules into chains (Fig. 2), in which they may be effective in the stabilization of the structure. The π–π contacts between the thiadiazole rings, Cg2···Cg2^i^ and Cg2···Cg1^ii^ [symmetry codes: (i) -x, 1 - y, -z; (ii) -x, -y, -z, where Cg1 and Cg2 are the centroids of the rings A (S1/N1/N2/C3/C4) and B (S2/N3/N4/C7/C8), respectively] may further stabilize the structure, with centroid–centroid distances of 3.699 (3) and 3.720 (2) Å, respectively.

### Experimental

For the preparation of the title compound, 5-amino-l,3,4-thiadiazole (5 mmol) was dissolved by heating in concentrated HCl (50 ml) in a water bath, after which the solution was cooled to 268 K and a solution of sodium nitrite (2.5 mmol) in water (3.5 ml) was added dropwise with stirring. The resulting bright-yellow diazonium solution was allowed to stand in ice for 30 min, after which a saturated solution of sodium acetate (100 ml, pH = 5) was added. The precipitate was removed by filtration, and purified by crystallization from toluene. Crystals suitable for X-ray analysis were obtained by slow evaporation of an ethanol solution.

### Refinement

H atoms were positioned geometrically, with N—H = 0.86 Å (for NH) and C—H = 0.97 and 0.96 Å for methylene and methyl H, respectively, and constrained to ride on their parent atoms with U_iso_(H) = xU_eq_(C,N), where x = 1.5 for methyl H and x = 1.2 for all other H atoms.

### Crystal data

**Table:**

C_8_H_11_N_7_S_2_	*F*(000) = 560
*M**_r_* = 269.36	*D*_x_ = 1.344 Mg m^−^^3^
Monoclinic, *P*2_1_/*c*	Mo *K*α radiation, λ = 0.71073 Å
Hall symbol: -P 2ybc	Cell parameters from 25 reflections
*a* = 12.188 (2) Å	θ = 9–12°
*b* = 9.1460 (18) Å	µ = 0.39 mm^−^^1^
*c* = 12.790 (3) Å	*T* = 294 K
β = 110.99 (3)°	Block, yellow
*V* = 1331.1 (5) Å^3^	0.20 × 0.10 × 0.10 mm
*Z* = 4	

### Data collection

**Table:**

Enraf–Nonius CAD-4 diffractometer	1468 reflections with *I* > 2σ(*I*)
Radiation source: fine-focus sealed tube	*R*_int_ = 0.005
graphite	θ_max_ = 25.3°, θ_min_ = 1.8°
ω/2θ scans	*h* = −14→13
Absorption correction: ψ scan (North *et al.*, 1968)	*k* = 0→10
*T*_min_ = 0.926, *T*_max_ = 0.962	*l* = 0→14
2431 measured reflections	3 standard reflections every 120 min
2320 independent reflections	intensity decay: none

### Refinement

**Table:**

Refinement on *F*^2^	Primary atom site location: structure-invariant direct methods
Least-squares matrix: full	Secondary atom site location: difference Fourier map
*R*[*F*^2^ > 2σ(*F*^2^)] = 0.072	Hydrogen site location: inferred from neighbouring sites
*wR*(*F*^2^) = 0.178	H-atom parameters constrained
*S* = 1.00	*w* = 1/[σ^2^(*F*_o_^2^) + (0.06*P*)^2^ + 3.61*P*] where *P* = (*F*_o_^2^ + 2*F*_c_^2^)/3
2320 reflections	(Δ/σ)_max_ < 0.001
142 parameters	Δρ_max_ = 0.62 e Å^−^^3^
0 restraints	Δρ_min_ = −1.06 e Å^−^^3^

### Special details

**Table:**

Geometry. All e.s.d.'s (except the e.s.d. in the dihedral angle between two l.s. planes) are estimated using the full covariance matrix. The cell e.s.d.'s are taken into account individually in the estimation of e.s.d.'s in distances, angles and torsion angles; correlations between e.s.d.'s in cell parameters are only used when they are defined by crystal symmetry. An approximate (isotropic) treatment of cell e.s.d.'s is used for estimating e.s.d.'s involving l.s. planes.
Refinement. Refinement of *F*^2^ against ALL reflections. The weighted *R*-factor *wR* and goodness of fit *S* are based on *F*^2^, conventional *R*-factors *R* are based on *F*, with *F* set to zero for negative *F*^2^. The threshold expression of *F*^2^ > σ(*F*^2^) is used only for calculating *R*-factors(gt) *etc*. and is not relevant to the choice of reflections for refinement. *R*-factors based on *F*^2^ are statistically about twice as large as those based on *F*, and *R*- factors based on ALL data will be even larger.

### Fractional atomic coordinates and isotropic or equivalent isotropic displacement parameters (Å^2^)

**Table:**

	*x*	*y*	*z*	*U*_iso_*/*U*_eq_	
S1	1.26701 (14)	0.49714 (19)	0.57391 (10)	0.0675 (5)	
S2	0.96258 (11)	0.18590 (15)	0.56999 (10)	0.0488 (4)	
N1	1.3578 (5)	0.6450 (6)	0.4508 (4)	0.0804 (16)	
N2	1.2634 (4)	0.5731 (5)	0.3737 (3)	0.0548 (11)	
N3	0.7840 (4)	0.0373 (5)	0.4403 (3)	0.0554 (12)	
N4	0.8301 (3)	0.1075 (5)	0.3694 (3)	0.0512 (11)	
N5	0.9719 (4)	0.2610 (5)	0.3544 (3)	0.0515 (11)	
H5A	0.9482	0.2556	0.2825	0.062*	
N6	1.1099 (3)	0.4124 (5)	0.3558 (3)	0.0463 (10)	
N7	1.0651 (3)	0.3422 (4)	0.4200 (3)	0.0417 (9)	
C1	1.5073 (6)	0.6346 (8)	0.7641 (5)	0.092	
H1B	1.5528	0.7039	0.8189	0.138*	
H1C	1.5570	0.5558	0.7586	0.138*	
H1D	1.4453	0.5968	0.7861	0.138*	
C2	1.4558 (5)	0.7082 (7)	0.6535 (5)	0.073	
H2A	1.5195	0.7411	0.6308	0.088*	
H2B	1.4135	0.7943	0.6626	0.088*	
C3	1.3715 (6)	0.6141 (9)	0.5586 (5)	0.090 (2)	
C4	1.2070 (4)	0.4920 (6)	0.4245 (4)	0.0466 (12)	
C5	0.7047 (6)	−0.0851 (8)	0.6080 (5)	0.0790 (19)	
H5B	0.6879	−0.1221	0.6709	0.119*	
H5C	0.7149	−0.1654	0.5640	0.119*	
H5D	0.6406	−0.0251	0.5629	0.119*	
C6	0.8141 (5)	0.0036 (7)	0.6486 (4)	0.0589 (14)	
H6B	0.8787	−0.0567	0.6950	0.071*	
H6C	0.8043	0.0837	0.6941	0.071*	
C7	0.8436 (4)	0.0651 (6)	0.5493 (4)	0.0509 (13)	
C8	0.9231 (4)	0.1912 (6)	0.4186 (4)	0.0465 (12)	

### Atomic displacement parameters (Å^2^)

**Table:**

	*U*^11^	*U*^22^	*U*^33^	*U*^12^	*U*^13^	*U*^23^
S1	0.0757 (10)	0.0889 (12)	0.0315 (7)	−0.0236 (9)	0.0116 (6)	0.0029 (7)
S2	0.0510 (7)	0.0616 (8)	0.0329 (6)	−0.0063 (7)	0.0140 (5)	−0.0044 (6)
N1	0.073 (3)	0.099 (4)	0.062 (3)	−0.029 (3)	0.016 (3)	0.013 (3)
N2	0.053 (3)	0.066 (3)	0.043 (2)	−0.006 (2)	0.015 (2)	0.011 (2)
N3	0.045 (2)	0.074 (3)	0.049 (3)	−0.006 (2)	0.019 (2)	−0.012 (2)
N4	0.044 (2)	0.074 (3)	0.037 (2)	−0.005 (2)	0.0164 (19)	−0.010 (2)
N5	0.052 (3)	0.069 (3)	0.033 (2)	0.004 (2)	0.014 (2)	0.002 (2)
N6	0.049 (2)	0.057 (3)	0.032 (2)	−0.002 (2)	0.0141 (19)	0.0018 (19)
N7	0.038 (2)	0.049 (2)	0.036 (2)	0.0040 (18)	0.0114 (18)	−0.0001 (18)
C1	0.092	0.092	0.092	0.000	0.033	0.000
C2	0.073	0.073	0.073	0.000	0.026	0.000
C3	0.083 (4)	0.126 (6)	0.047 (3)	−0.045 (4)	0.008 (3)	0.010 (4)
C4	0.047 (3)	0.059 (3)	0.034 (2)	0.007 (3)	0.015 (2)	0.005 (2)
C5	0.084 (4)	0.095 (5)	0.065 (4)	−0.030 (4)	0.035 (3)	−0.004 (4)
C6	0.067 (3)	0.072 (4)	0.044 (3)	−0.019 (3)	0.029 (3)	−0.012 (3)
C7	0.048 (3)	0.061 (3)	0.043 (3)	−0.004 (3)	0.015 (2)	−0.010 (2)
C8	0.045 (3)	0.053 (3)	0.035 (2)	0.001 (2)	0.007 (2)	−0.006 (2)

### Geometric parameters (Å, °)

**Table:**

S1—C3	1.728 (6)	C1—H1B	0.9600
S1—C4	1.786 (5)	C1—H1C	0.9600
S2—C7	1.766 (5)	C1—H1D	0.9600
S2—C8	1.820 (5)	C2—C3	1.541 (8)
N1—N2	1.384 (6)	C2—H2A	0.9700
N1—C3	1.358 (7)	C2—H2B	0.9700
N2—C4	1.328 (6)	C4—N6	1.399 (6)
N3—N4	1.384 (6)	C5—C6	1.487 (7)
N3—C7	1.346 (6)	C5—H5B	0.9600
N4—C8	1.325 (6)	C5—H5C	0.9600
N5—N7	1.365 (5)	C5—H5D	0.9600
N5—C8	1.338 (6)	C6—C7	1.544 (7)
N5—H5A	0.8600	C6—H6B	0.9700
N6—N7	1.306 (5)	C6—H6C	0.9700
C1—C2	1.486 (7)		
			
C3—S1—C4	86.0 (3)	N1—C3—S1	114.6 (5)
C7—S2—C8	88.1 (2)	C2—C3—S1	124.5 (5)
C3—N1—N2	113.1 (5)	N2—C4—N6	116.9 (4)
C4—N2—N1	111.2 (4)	N2—C4—S1	115.1 (4)
C7—N3—N4	113.3 (4)	N6—C4—S1	128.0 (4)
C8—N4—N3	115.8 (4)	C6—C5—H5B	109.5
N7—N5—H5A	125.1	C6—C5—H5C	109.5
C8—N5—N7	109.7 (4)	H5B—C5—H5C	109.5
C8—N5—H5A	125.1	C6—C5—H5D	109.5
N7—N6—C4	108.2 (4)	H5B—C5—H5D	109.5
N6—N7—N5	108.9 (4)	H5C—C5—H5D	109.5
C2—C1—H1B	109.5	C5—C6—C7	110.8 (4)
C2—C1—H1C	109.5	C5—C6—H6B	109.5
H1B—C1—H1C	109.5	C7—C6—H6B	109.5
C2—C1—H1D	109.5	C5—C6—H6C	109.5
H1B—C1—H1D	109.5	C7—C6—H6C	109.5
H1C—C1—H1D	109.5	H6B—C6—H6C	108.1
C1—C2—C3	115.6 (6)	N3—C7—C6	125.8 (5)
C1—C2—H2A	108.4	N3—C7—S2	112.5 (4)
C3—C2—H2A	108.4	C6—C7—S2	121.7 (4)
C1—C2—H2B	108.4	N4—C8—N5	118.5 (4)
C3—C2—H2B	108.4	N4—C8—S2	110.2 (4)
H2A—C2—H2B	107.4	N5—C8—S2	131.3 (4)
N1—C3—C2	119.3 (6)		
			
C3—N1—N2—C4	1.1 (8)	N4—N3—C7—C6	179.9 (5)
N2—N1—C3—C2	−167.6 (6)	N4—N3—C7—S2	0.9 (6)
N2—N1—C3—S1	−1.5 (9)	C5—C6—C7—N3	−3.6 (8)
C1—C2—C3—N1	−153.7 (7)	C5—C6—C7—S2	175.3 (4)
C1—C2—C3—S1	41.7 (9)	C8—S2—C7—N3	−0.6 (4)
C4—S1—C3—N1	1.1 (6)	C8—S2—C7—C6	−179.6 (5)
C4—S1—C3—C2	166.4 (7)	N3—N4—C8—N5	179.6 (4)
C7—N3—N4—C8	−0.9 (6)	N3—N4—C8—S2	0.4 (5)
N1—N2—C4—N6	−179.5 (4)	N7—N5—C8—N4	179.4 (4)
N1—N2—C4—S1	−0.3 (6)	N7—N5—C8—S2	−1.6 (6)
C3—S1—C4—N2	−0.5 (5)	C7—S2—C8—N4	0.1 (4)
C3—S1—C4—N6	178.7 (5)	C7—S2—C8—N5	−178.9 (5)
N2—C4—N6—N7	−178.9 (4)	C4—N6—N7—N5	−178.5 (4)
S1—C4—N6—N7	1.9 (6)	C8—N5—N7—N6	−178.4 (4)

### Hydrogen-bond geometry (Å, °)

**Table:**

*D*—H···*A*	*D*—H	H···*A*	*D*···*A*	*D*—H···*A*
N5—H5A···S2^i^	0.86	2.84	3.631 (4)	154

Symmetry codes: (i) *x*, −*y*+1/2, *z*−1/2.
